# TurboFlow在线净化-液相色谱-串联质谱法快速检测人尿中鹅膏肽类毒素

**DOI:** 10.3724/SP.J.1123.2020.06005

**Published:** 2021-03-08

**Authors:** Li FANG, Fengmei QIU, Xinwei YU

**Affiliations:** 1.浙江省海产品健康危害因素关键技术研究重点实验室(舟山市疾病预防控制中心), 浙江 舟山 316021; 1. Key Laboratory of Health Risk Factors for Seafood of Zhejiang Province (Zhoushan Municipal Center for Disease Control and Prevention), Zhoushan 316021, China; 2.岱山县疾病预防控制中心, 浙江 岱山 316200; 2. Daishan Center for Disease Control and Prevention, Daishan 316200, China

**Keywords:** 在线净化, 液相色谱-串联质谱法, 鹅膏肽类毒素, 蘑菇毒素, 尿液, online-cleanup, liquid chromatography-tandem mass spectrometry (LC-MS/MS), amanita peptide toxins, mushroom toxins, urine

## Abstract

鹅膏肽类毒素是一类环状多肽类蘑菇毒素,中毒后会造成急性肝损伤,病死率非常高。我国因误食野生毒蘑菇导致的中毒事件常有发生,测定人尿中鹅膏肽类毒素的浓度,可为临床早期诊断和救治提供有价值的信息。该研究建立了TurboFlow (TF)在线净化-液相色谱-三重四极杆质谱快速定量检测尿液中5种鹅膏肽类毒素(*α*-鹅膏毒肽、*β*-鹅膏毒肽、*γ*-鹅膏毒肽、羧基二羟鬼笔毒肽和二羟鬼笔毒肽)的新方法。尿液样品经高速离心后,直接注入TurboFlow在线净化-液相色谱-串联质谱进行分析。对影响TF在线净化的参数如TF净化柱、上样溶剂、洗脱溶剂、转移流速、转移时间等进行了优化。在优化后的实验条件下,以TurboFlow^TM^Cyclone柱(50 mm×0.5 mm)为净化柱,Hypersil GOLD C_18_柱(100 mm×2.1 mm)为分析柱,甲醇和4 mmol/L乙酸铵为流动相进行梯度洗脱,电喷雾正离子选择反应监测(SRM)模式下进行检测,基质匹配外标法定量。结果表明,鹅膏肽类毒素在1.0~50.0 μg/L范围内呈现良好的线性关系,相关系数均可达到0.997以上。方法的检出限为0.15~0.3 μg/L,定量限为0.5~1.0 μg/L。在2.0、5.0和10.0 μg/L的加标水平下,5种鹅膏肽类毒素的日内和日间回收率分别为87.0%~108.6%和86.8%~112.7%,日内、日间相对标准偏差(RSD)均小于14.5%。该检测方法准确、快速、灵敏度高、易操作,适用于公共卫生应急检测或临床中毒病因识别检测。

## Abstract (en)

Amanita peptide toxins are cyclic polypeptide mushroom toxins that can cause acute liver damage. The fatality rate associated with these toxins is very high. Monitoring the concentration of amanita peptide toxins in human urine can provide valuable information for early clinical diagnosis and treatment. Therefore, a TurboFlow online clean-up-liquid chromatography-triple quadrupole mass spectrometry (TF-LC-MS/MS) method was established for the simultaneous quantitative determination of five amanita peptide toxins (*α*-amanitin, *β*-amanitin, *γ*-amanitin, phallacidin, and phalloidin) in human urine. After pre-treatment with high-speed centrifugation, urine samples were analyzed using TF-LC-MS/MS. The main factors influencing purification efficiency, including the TF column, loading solution, eluting solution, transfer flow, and transfer time, were optimized in this study. Under the optimized experimental conditions, the analytes were purified using a TurboFlow^TM^ Cyclone column (50 mm×0.5 mm) and separated on a Hypersil GOLD C_18_ column (100 mm×2.1 mm) using the mobile phases of methanol and 4 mmol/L aqueous ammonium acetate solution with gradient elution. The analytes were detected in selected reaction monitoring (SRM) mode via positive electrospray ionization. Matrix-matched external standard calibration was used for quantitation. The linear range of the method ranged from 1.0 μg/L to 50.0 μg/L for all five amanita peptide toxins, with correlation coefficients (*r*^2^) higher than 0.997. The limits of detection were 0.15-0.3 μg/L and the limits of quantification (LOQs) were 0.5-1.0 μg/L for the five amanita peptide toxins in urine. The intra-day and inter-day recoveries of amanita peptide toxins were 87.0%-108.6% and 86.8%-112.7%, respectively, at the spiked levels of 2.0, 5.0, and 10.0 μg/L. The intra-day and inter-day relative standard deviations (RSDs) were less than 14.5%. The method is accurate, rapid, sensitive, easy to operate, and can satisfy the requirements of public health emergency testing or clinical poisoning testing.

我国山区众多,野生菌类资源丰富、种类繁多,因误食野生毒蘑菇中毒事件时有发生,并可引起严重甚至致命的中毒。鹅膏肽类毒素是一类环状多肽类毒素,由该毒素引起的中毒占毒蘑菇中毒死亡病例的90%以上^[1]^。根据氨基酸的组成和结构,常见鹅膏肽类毒素分为鹅膏毒肽、鬼笔毒肽和毒伞肽三大类。鹅膏毒肽为双环八肽,主要包括*α*-鹅膏毒肽(*α*-amanitin)、*β*-鹅膏毒肽(*β*-amanitin)和*γ*-鹅膏毒肽(*γ*-amanitin);鬼笔毒肽为双环七肽,主要包括羧基二羟鬼笔毒肽(phallacidin)和二羟鬼笔毒肽(phalloidin);毒伞素为单环七肽,致命性相对较弱,相关研究较少^[2]^。鹅膏肽类毒素具有强烈的肝毒性,其中鹅膏毒肽的口服半数致死剂量(LD_50_)可达0.1 mg/kg,单个毒蘑菇中亦可能含有该毒素剂量;鬼笔毒肽是一种速效毒素,能专一性地与细胞中的肌丝蛋白结合,进而打破肌丝蛋白与肌球蛋白之间聚合和解聚的动态平衡,动物静脉或腹腔注射实验,2~5 h内死亡^[3]^。

鹅膏肽类毒素引起的蘑菇中毒往往有一定的潜伏期,一般为6~24 h,在极端情况下最长可达36 h,然后才出现临床中毒症状^[4]^。有研究表明毒蘑菇摄取后约30 h内血清中可检测到鹅膏毒肽;摄取后约72 h内尿液中可检测到鹅膏毒肽^[5]^。因此,与血液样本相比,尿液样本更适合用于鹅膏类蘑菇毒素中毒事件检测。有文献报道最早在毒蘑菇摄入后90 min就发现尿液中存在毒素^[6]^。尽管鹅膏肽类毒素在毒蘑菇中浓度很高,但生物样品中相关毒素的检出浓度非常低,在μg/L甚至更低水平^[3,7]^。因此,开发建立一种高灵敏度、快速定量检测尿液中鹅膏肽类毒素的方法也显得十分必要。

目前,关于尿液中鹅膏肽类毒素的检测方法主要有酶联免疫法^[8]^、高效液相色谱法^[6,9]^、毛细管电泳法^[10]^及液相色谱-串联质谱法^[3,5,11-15]^。酶联免疫法是一种快速筛选方法;液相色谱、毛细管电泳配合紫外或二极管阵列检测器存在检测灵敏度相对较低的问题;液相色谱-串联质谱法是目前的主流方法,具有选择性好、灵敏度高等优势。当前关于生物样品中鹅膏肽类毒素残留检测的前处理手段主要有蛋白质沉淀^[11]^、固相萃取^[3,5,12-14]^、免疫亲和提取法^[15]^等。常规固相萃取小柱净化法存在溶剂消耗量大、使用成本高等缺陷;蛋白质沉淀法存在净化效果差等缺陷,这些前处理方法均在离线状态下进行,费时费力。与传统的净化方法相比,TurboFlow (TF)在线净化通过扩散溶解、尺寸排阻等技术将蛋白质等生物大分子物质过滤掉,保留目标小分子化合物;并通过与串联质谱联用大大简化样品前处理过程,实现在线净化功能的同时,也保证了检测方法的灵敏度^[16,17]^。目前该技术已应用于尿液中*β*-激动剂^[18]^、全氟化合物^[19]^、多环芳烃代谢物^[20]^、紫外线吸收剂^[21]^等典型污染物的检测。Helfer等^[13]^首先利用TF在线净化技术,开展尿液中鹅膏毒肽类的检测,但该研究采用两根TurboFlow柱串联净化,增加了使用成本,且未涉及鬼笔毒肽的检测。徐小民等^[22]^采用在线ODS微柱结合单六通阀切换技术,实现了尿液中痕量*α*-鹅膏毒肽的检测,也未涉及鬼笔毒肽的检测。魏佳会等^[3]^报道毒蘑菇中毒病人尿液样本中存在鬼笔毒肽类毒素。

本研究利用TF-LC-MS/MS系统,建立了直接进样快速测定尿液中3种鹅膏毒肽和2种鬼笔毒肽的检测方法。该方法简便、快速、结果准确可靠,不需要复杂前处理,可用于中毒病人尿液的快速定性定量分析,为判定公共卫生中毒事件原因和临床准确识别毒蘑菇中毒提供了技术支持。

## 1 实验部分

### 1.1 仪器与试剂

Transcend Ⅱ在线净化-液相色谱系统(美国Thermo公司); TSQ Vantage三重四极杆质谱仪(美国Thermo公司); Microfuge 20R型微量高速冷冻离心机(美国Beckman公司); Milli-Q(18.2 MΩ)超纯水处理系统(德国Merck公司)。

甲酸(LC-MS级)、乙酸铵(色谱纯)、乙腈(色谱纯)购自德国CNW公司;超纯水由Milli-Q超纯水系统制得。*α*-鹅膏毒肽、*β*-鹅膏毒肽、*γ*-鹅膏毒肽、羧基二羟鬼笔毒肽和二羟鬼笔毒肽标准品购自美国Alexis公司,标准纯度≥90%。尿液由健康志愿者提供。

### 1.2 样品制备

取充分混合后的尿样1 mL于2 mL聚丙烯离心管中,在4 ℃条件下,以15000 r/min的转速离心10 min,取适量上清液于进样瓶中。

### 1.3 标准溶液的配制

鹅膏肽类毒素标准溶液用甲醇溶液稀释,配成质量浓度为1.0 μg/mL的标准储备液。用0.1%(v/v, 下同)甲酸水溶液配制成1、2、5、10、20和50 μg/L的纯溶剂标准系列;用空白尿液基质配制成为1、2、5、10、20和50 μg/L的尿液基质标准系列。

### 1.4 TF净化及色谱分离条件

富集净化柱:TurboFlow^TM^ Cyclone色谱柱(50 mm×0.5 mm, 60 μm),上样泵的流动相A为0.1%甲酸水溶液,流动相B为甲醇,流动相C为水,流动相D为乙腈,进样量为50 μL,在线净化梯度洗脱程序见表1(Loading pump), TF连接图见图1。

**表1:** 在线净化与色谱分离梯度洗脱程序

Step No.	Start time/ min	Step time/ min	Function	Loading pump						Eluting pump			
				Flow rate/ (mL/min)	*φ*(0.1% FA)/%	*φ*(MeOH)/ %	*φ*(H_2_O)/ %	*φ*(ACN)/ %		Flow rate/ (mL/min)		*φ*(4 mmol/L CH_3_COONH_4_)/%	*φ*(MeOH)/ %
1	0.0	1.0	loading	1.5	100	0	0	0		0.3		95	5
2	1.0	1.0	transferring	0.1	80	0	0	20		0.2		95	5
3	2.0	2.0	washing	1.5	0	0	90	10		0.3		80	20
4	4.0	1.5	washing	1.5	0	0	0	100		0.3		60	40
5	5.5	1.0	washing	1.5	0	100	0	0		0.3		40	60
6	6.5	0.5	washing	1.5	0	0	0	100		0.3		10	90
7	7.0	2.0	filling the loop	1.5	80	0	0	20		0.3		10	90
8	9.0	0.2	equilibrating	1.5	100	0	0	0		0.3		95	5
9	9.2	1.8	equilibrating	1.5	100	0	0	0		0.3		95	5

FA: formic acid aqueous solution.

**图1:**
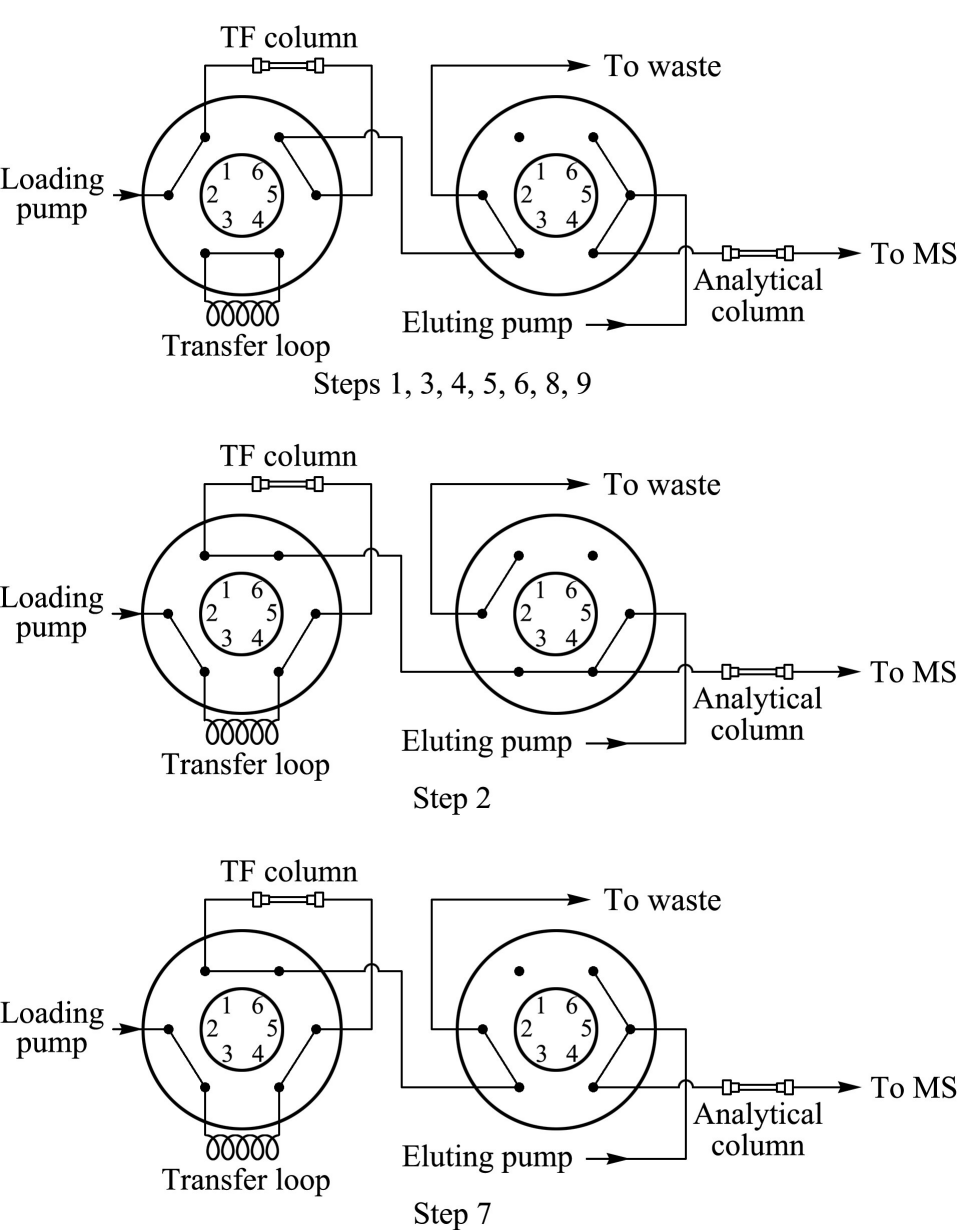
在线净化流路连接示意图

分析柱:Hypersil GOLD C_18_色谱柱(100 mm×2.1 mm, 1.9 μm,美国Thermo公司),流动相A为4 mmol/L乙酸铵水溶液,B为甲醇,流速为0.3 mL/min,梯度洗脱见表1(Eluting pump)。

### 1.5 质谱条件

电喷雾离子源(ESI),正离子扫描方式,选择反应监测(SRM)模式;喷雾电压为3.0 kV;汽化温度300 ℃;离子传输毛细管温度为325 ℃;鞘气压(sheath units)为45 arb,辅助气压(arbitrary units)为15 arb,这两种雾化气均为高纯氮气,碰撞气为高纯氩气,压力为0.2 Pa(1.5 mTorr)。使用前调节各气体流量以使质谱灵敏度达到检测要求。5种鹅膏肽类毒素的检测参数见表2。

**表2:** 鹅膏肽类毒素的质谱检测参数

Analyte	Scan mode	Precursor ion (*m/z*)	Product ion (*m/z*)	S-lens/ V	CE/ eV
*α*-Amanitin	ESI^+^	919.6	259.0^*^/86.0	180	33/48
*β*-Amanitin	ESI^+^	920.6	259.0^*^/86.0	180	32/46
*γ*-Amanitin	ESI^+^	903.6	243.0^*^/86.0	180	37/54
Phallacidin	ESI^+^	847.6	157.0^*^/329.9	160	50/28
Phalloidin	ESI^+^	789.6	157.0^*^/330.0	160	45/33

* Quantitative ion.

## 2 结果与讨论

### 2.1 色谱、质谱条件优化

国内外很多文献已证实反相色谱柱适合鹅膏肽类毒素的分离与检测^[3,5,14,15]^。本实验采用由4 mmol/L乙酸铵与甲醇组成的流动相体系,比较了Hypersil GOLD C_18_(100 mm×2.1 mm, 1.9 μm)和Acquity UPLC BEH C_18_ (100 mm×2.1 mm, 1.7 μm)两根小粒径反相色谱柱对目标物的色谱分离效果的影响。结果显示,两根C_18_柱均能较好地实现5种毒素的分离,但是Hypersil GOLD C_18_产生的系统压力明显低于Acquity UPLC BEH C_18_。因此本研究采用Hypersil GOLD C_18_柱分离待测组分,能较好地实现目标分析物的色谱分离,见图2。

**图2:**
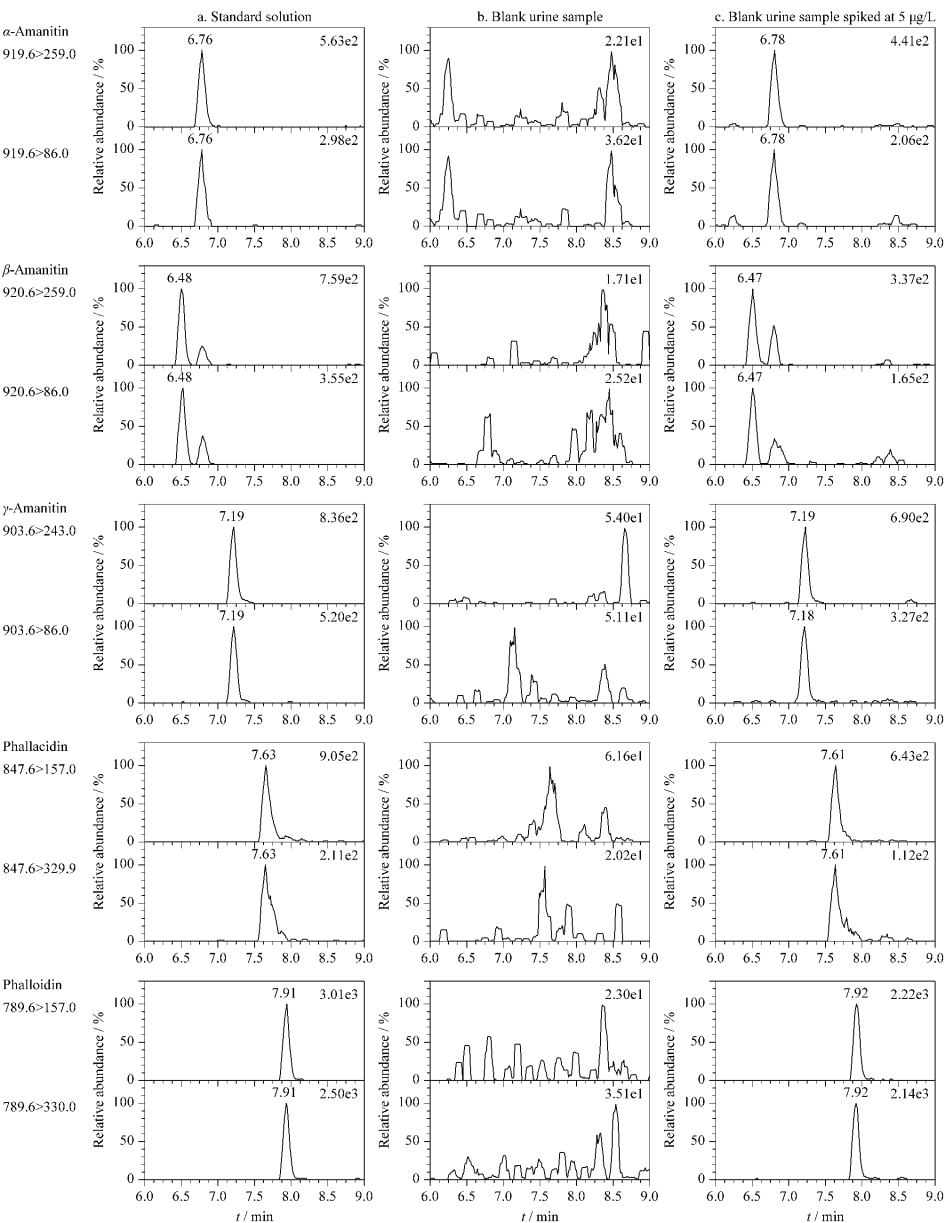
标准溶液、空白尿样及加标尿样中5种鹅膏肽类毒素的SRM色谱图

实验中分别取1.0 mg/L鹅膏肽类毒素标准溶液,以流动注射方式在电喷雾正离子模式下优化目标毒素的质谱参数,选择干扰小、信噪比大的离子对作为定性或定量离子对。优化后的透镜电压、碰撞能量,选择的定性和定量离子见表2。鉴于目标毒素的定量、定性子离子质量数较小(见表2),本研究考察了空白尿样对目标物定量、定性离子通道的影响。从图2b可见,所选的定量、定性离子通道在目标物出峰时间附近未见明显色谱峰干扰,各离子通道本底基线也相对较低。

### 2.2 在线净化条件优化

TF在线净化程序一般包括上样与净化、洗脱、LC分离、色谱柱淋洗与再生等步骤。需要对TF净化柱、上样溶剂、洗脱溶剂、转移流速和转移时间等参数进行优化。

2.2.1 TF净化柱的选择

为了选择最佳的在线TF净化柱,考察了3种具有不同化学修饰基团的TF净化柱。本研究选择了两种聚合物级的TF净化柱Cyclone(50 mm×0.5 mm)和Cyclone-P(50 mm×0.5 mm)和一种硅胶基TF净化柱XL-C_18_(50 mm×0.5 mm)。在最佳液相色谱条件下,将5种毒素混合标准溶液注入TF净化柱中,比较目标物在色谱柱上的保留能力。结果发现,Cyclone柱对5种目标毒素的保留效果最好。TF净化柱可重复使用,实际进样100针后目标物的色谱峰响应未见明显降低。

2.2.2 上样溶剂的选择

上样溶剂的组成会影响目标毒素在TF净化柱上的保留能力。本研究比较了0.1%甲酸水溶液和0.1%甲酸与甲醇组成的不同比例混合溶液作为上样溶液对目标毒素的色谱峰强度的影响。从图3a中可见,尽管上样液中含有机溶剂有助于从样品中去除不需要的杂质,但随着上样溶液中有机相甲醇含量的不断增加,目标毒素的色谱峰强度随之不断下降,这表明有机相甲醇的存在,降低了目标毒素在TF柱的保留,因此本研究采用0.1%甲酸水溶液作为上样溶液。

**图3:**
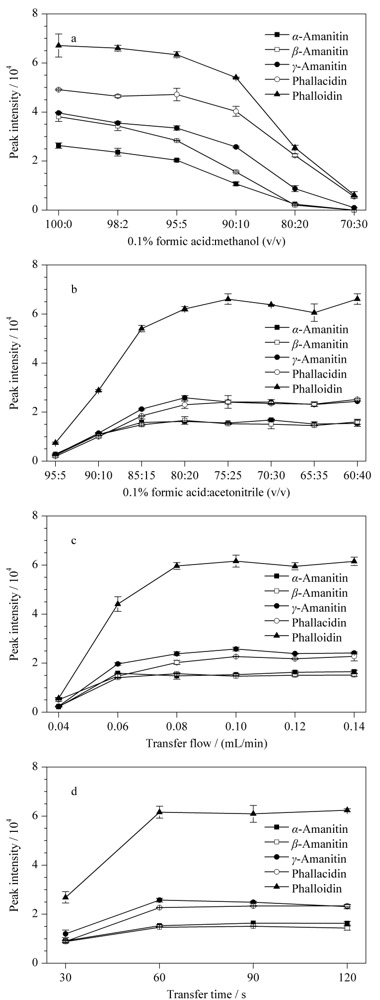
在线净化条件对目标物色谱峰响应的影响(*n*=3)

2.2.3 洗脱溶剂的选择

高有机强度洗脱溶剂可在TF净化柱上获得令人满意的分析物回收率,但在分析柱上无法产生尖锐的色谱峰。因此,对洗脱溶剂进行了优化,以同时实现目标物的定量洗脱和所需的峰锐度。本研究考察了乙腈和0.1%甲酸组成的不同比例混合物作为洗脱溶剂对目标毒素色谱峰强度的影响。由图3b可见,当乙腈的比例从5%变为20%时,5种目标毒素的回收率随乙腈比例的上升而升高。当洗脱溶剂中乙腈比例超过20%时,目标毒素的回收率未见明显升高。研究结果表明20%的乙腈足以完全将目标物从TF净化柱上洗脱下来,且目标毒素色谱峰形尖锐对称。因此,选择乙腈-0.1%甲酸(20∶80, v/v)作为洗脱溶剂。

2.2.4 转移流速的选择

转移步骤就是将分析物从TF柱转移洗脱到分析柱。较低的转移洗脱流速可能导致峰展宽和保留时间更长。较高的转移流速可加快分析物的转移,但可能会增加色谱柱的压力。在优化实验中,在转移时间为60 s的条件下,比较了转移流速0.06、0.08、0.10、0.12和0.14 mL/min对5种目标毒素峰强度的影响。从图3c可见,当传输流速为0.06 mL/min,目标毒素色谱峰强度非常小,这表明目标物转移不完全,部分目标物仍保留在TF净化柱。随着转移流速的不断增加,峰面积也随之增加,当转移流速到达0.10 mL/min后,目标物的峰强度未出现显著增加。这表明当转移流速到达0.10 mL/min时,目标物已完全从TF柱转移至分析柱。因此,最优转移流速设定为0.10 mL/min。

2.2.5 转移时间的选择

转移时间和转移流速都决定了目标物的洗脱完全与否。本研究在0.10 mL/min的转移流速下,测试了30、60、90和120 s的转移时间对目标毒素的色谱峰响应的影响。如图3d所示,转移时间为30 s时,5种鹅膏肽类毒素的色谱峰强度均很小,这表明目标物转移不完全。当转移时间为60 s时,鹅膏肽类毒素转移到分析柱的比例已超过90%,且色谱峰形良好。当转移时间增加为90或120 s时,目标毒素的峰强度虽略有提高,但是更长的转移时间将导致更长的分析时间,且更多杂质将随目标毒素一起洗脱出来。因此,本研究选择60 s为最佳转移时间。

### 2.3 基质效应评估

本研究采用基质匹配标准曲线的斜率与纯溶剂标准曲线斜率的百分比来评价检测方法基质效应(matrix effect, ME)。评价可按(基质匹配标准曲线斜率/纯溶剂标准曲线斜率-1)×100%公式进行。低于100%表示存在基质抑制效应,高于100%表示存在基质增强效应,绝对值越大则基质效应越强,如恰好为100%则表示不存在基质效应。从表3可知,鹅膏类毒素在尿液基质中均呈现一定的基质抑制作用,基质效应在-31.6%~-52.7%之间,采用基质匹配标准曲线测定实际样品,可在一定程度上弥补基质效应对目标毒素定量检测的影响。

**表3:** 5种鹅膏肽类毒素的回归方程、相关系数、基质效应、检出限和定量限

Toxin	Linear range/(μg/L)	Solvent			Urine					
		Curve	*R* ^2^		Curve	*R* ^2^		ME/%	LOD/(μg/L)	LOQ/(μg/L)
*α*-Amanitin	1.0-50.0	*y*=918.615*x* -191.188	0.9994		*y*=578.167*x*-298.264	0.9990		-37.1	0.3	1.0
*β*-Amanitin	1.0-50.0	*y*=1018.51*x*-34.4314	0.9970		*y*=481.957*x*-61.4861	0.9998		-52.7	0.3	1.0
*γ*-Amanitin	1.0-50.0	*y*=1371.64*x* -126.402	0.9996		*y*=915.107*x*-172.278	0.9990		-33.3	0.3	1.0
Phallacidin	1.0-50.0	*y*=1794.66*x*-587.165	0.9994		*y*=942.94*x*-126.634	0.9999		-47.5	0.3	1.0
Phalloidin	1.0-50.0	*y*=4240.93*x*+3.11143	0.9992		*y*=2899.95*x*+548.874	0.9995		-31.6	0.15	0.5

ME=(the slope of matrix-matched standard curve/the slope of solvent standard curve-1)×100%; *y*: peak area of amanita peptide toxin; *x*; mass concentration of amanita peptide toxin.

### 2.4 标准曲线及检出限

按1.3节方法配制标准溶液并测定,以鹅膏肽类毒素的质量浓度为横坐标,目标物峰面积为纵坐标,绘制基质标准曲线,在1.0~50 μg/L浓度范围,线性相关性良好,相关系数均大于0.997。在本实验条件下,以3倍信噪比所对应的质量浓度为检出限,10倍信噪比所对应的质量浓度为定量限,得出尿液基质中二羟鬼笔毒肽的方法检出限和定量限分别为0.15 μg/L和0.5 μg/L,其他鹅膏肽类毒素的检出限和定量限分别可达到0.3 μg/L和1.0 μg/L。结果见表3。

### 2.5 方法的回收率与精密度

本研究以尿液为研究对象,考察检测方法的回收率和精密度。准确移取空白尿液1.0 mL,设定3个添加水平2.0、5.0和10.0 μg/L,根据上述方法进行检测,每组样品6个平行,连续测试3天,测定日内、日间回收率以及日内、日间相对标准偏差(RSD)。由表4可知,尿液基质中鹅膏肽类毒素的日内和日间回收率分别为87.0%~108.6%和86.8%~112.7%;日内、日间RSD均小于为14.5%,可满足检测分析的要求。

**表4:** 方法的回收率和精密度

Toxin	Spiked level/ (μg/L)	Intra-day (*n*=6)			Inter-day (*n*=3)	
		Recovery/ %	RSD/ %		Recovery/ %	RSD/ %
*α*-Amanitin	2.0	101.5	9.8		107.1	5.5
	5.0	100.5	7.4		106.0	4.3
	10.0	102.9	5.6		104.4	1.9
*β*-Amanitin	2.0	95.4	14.2		97.3	5.5
	5.0	89.2	4.0		94.4	4.3
	10.0	99.2	7.8		94.6	4.6
*γ*-Amanitin	2.0	108.6	13.6		112.7	7.2
	5.0	98.1	7.7		100.4	2.5
	10.0	95.3	6.4		96.7	2.9
Phallacidin	2.0	96.0	14.5		93.5	5.6
	5.0	91.7	6.5		89.4	5.7
	10.0	93.0	4.0		87.5	4.0
Phalloidin	2.0	90.2	5.7		87.9	2.3
	5.0	87.0	2.7		86.8	2.1
	10.0	89.1	4.0		88.0	0.8

## 3 结论

本方法采用TurboFlow在线净化,高效液相色谱-三重四极杆质谱检测,实现了快速定性定量检测尿液中3种鹅膏毒肽和2种鬼笔毒肽。本方法检测灵敏度高、重现性好、不需要复杂前处理,总运行时间仅为11 min,适用于毒蘑菇中毒事件快速筛查和临床中毒病因识别。
